# Elucidating the Magnetoelastic Coupling, Pressure-Dependent Magnetic Behavior, and Anomalous Hall Effect in Fe_*x*_Ti_2_S_4_ Intercalation Sulfides

**DOI:** 10.1021/acsami.3c12571

**Published:** 2023-10-20

**Authors:** Romualdo S. Silva, João E. Rodrigues, Angelika D. Rosa, Javier Gainza, Eva Céspedes, Norbert M. Nemes, José L. Martínez, José A. Alonso

**Affiliations:** † Instituto de Ciencia de Materiales de Madrid (ICMM), CSIC, E-28049 Madrid, Spain; ‡ European Synchrotron Radiation Facility (ESRF), 71 Avenue des Martyrs, 38000 Grenoble, France; § Departamento Física de Materiales, Universidad Complutense de Madrid, E-28040 Madrid, Spain

**Keywords:** high-pressure synthesis, sulfides, X-ray-absorption spectroscopy, hydrostatic pressure, magnetoelastic coupling, magnetoresistance, anomalous Hall effect

## Abstract

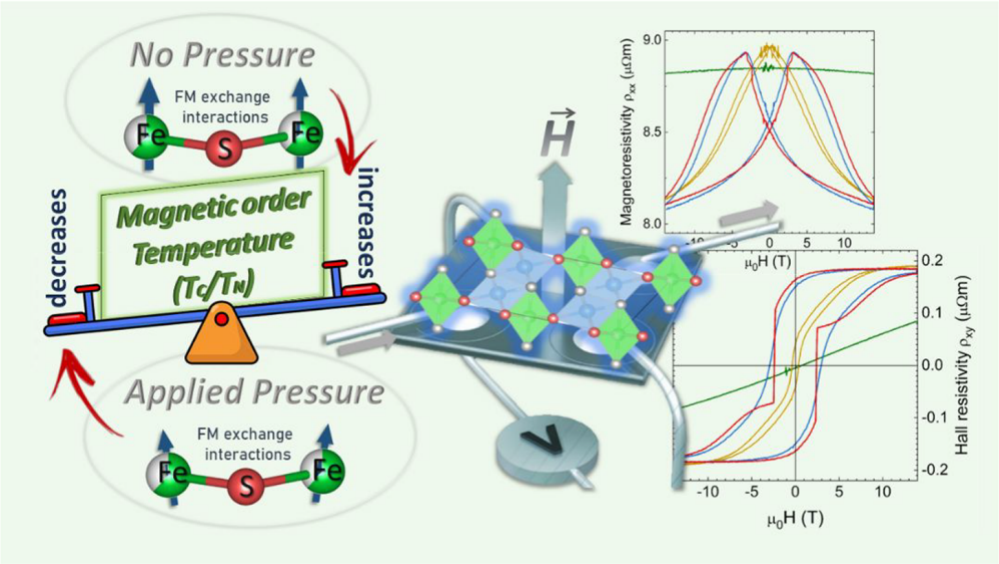

Transition-metal chalcogenides with intercalated layered structures are interesting systems in material physics due to their attractive electronic and magnetic properties, with applications in the fields of magnetic refrigerators, catalysts, and thermoelectrics, among others. In this work, we studied in detail the structural, electronic, and magnetic properties of (Fe,Ti)-based sulfides with formula Fe_*x*_Ti_2_S_4_ (*x* = 0.24, 0.32, and 0.42), prepared as polycrystalline materials under high-pressure conditions. They present a layered Heideite-type crystal structure, as assessed by synchrotron X-ray diffraction. A local structure analysis using Fe *K*-edge extended X-ray-absorption fine structure (EXAFS) data unveiled a conspicuous contraction of the main Fe–S bond in Fe_0.24_Ti_2_S_4_ at the vicinity of the magnetic transition 60–80 K. We suggest that this anomaly is related to magnetoelastic coupling effects. The EXAFS analysis allowed extraction of the Einstein temperatures (θ_*E*_), i.e., the phonon contribution to the specific heat, for the two bond pairs Fe–S_(1)_ [θ_*E*_ ≈318 K; 290 K (*C*/*T*)] and Fe–Ti_(1)_ [θ_*E*_ ≈218 K; 190 K (*C*/*T*)]. In addition to the structural and local vibrational measurements, we probed the magnetic properties using magneto-calorimetry, magnetometry under applied pressure, magnetoresistance (MR), and Hall effect measurements. We observed the appearance of a broad peak in the specific heat around 120 K in the *x* = 0.42 compound that we associated with an antiferromagnetic ordering electronic transition. We found that the antiferromagnetic transition temperature is pressure and composition sensitive and reduces at 1.2 GPa by ∼12 and ∼3 K, for the members with *x* = 0.24 and *x* = 0.42, respectively. Similarly, the saturation magnetization in the ordered phase depends on both pressure and iron content, reducing its value by 50, 90, and 30% for *x* = 0.24, 0.32, and 0.42, respectively. We observed clear jumps in the magnetic hysteresis loops, MR, and anomalous Hall effect (AHE) below 2 K at fields around 2–4 T. We associated this observation with the metamagnetic transitions; from the Berry-curvature a decoupling parameter of *S*_H_ = 0.12 V^–1^ is determined. Comparison of the results on the temperature-dependent magnetization, MR, and AHE elucidates a strong inelastic scattering contribution to the AHE at higher temperatures due to the cluster spin-glass phase.

## 1 Introduction

Transition metal chalcogenides have been the subject of numerous studies due to their unique properties and potential interest in the modern high-tech industry, such as transistors,^1^ photodetectors,^2,3^ electroluminescent devices,^4^ catalysis,^5^ energy conversion,^6^ thermoeletric materials,^7,8^ magnetic refrigeration,^9^ and various other electronic devices.^10,11^ For instance, the thiospinel compounds (Hg,Cd,Fe)Cr_2_S_4_ show coexisting colossal magnetoresistance (MR) and magnetocapacitance effects.^12^ Beyond 3D crystal structures, like spinels, perovskites, etc., intercalation metal sulfides M_*x*_B_2_S_4_ (M, B: transition metals) are attracting much attention for technological applications due to their enhanced electrical properties and richer redox chemistry than the traditional binary metal disulfides (*BS*_2_). The *M*^2+^ atoms insertion into *BS*_2_ layers significantly drives physical properties such as the magnetoelectric coupling, large MR effects, colossal magnetocapacitive effects, magnetocaloric effects, etc. Within this family of intercalation sulfides, in Fe_*x*_Ta_2_S_4_ ferromagnets, an anomalous Hall effect (AHE) was discovered recently.^13^ For the Fe_*x*_Ti_2_S_4_ system, we recently demonstrated the cluster spin-glass (CSG) magnetic behavior and magnetocaloric properties.^9^ The crystal structure of these intercalation compounds consists of layers of edge-sharing TiS_6_ octahedra, with Fe atoms located between the layers, also in octahedral coordination. Due to the structural similarities between Fe_*x*_Ta_2_S_4_ and Fe_*x*_Ti_2_S_4_, the present work aims at demonstrating and better understanding the AHE in the latter sulfides and its dependence on the composition. Our results may prove that the AHE is strongly dependent on the iron intercalation level and its crystalline environment.

Polycrystalline Fe_*x*_Ti_2_S_4_ materials with Fe contents below 1 (*x* < 1) exhibit a metallic-like resistivity behavior at temperatures above 150 K. For higher Fe contents, with *x* > 1, the resistivity in the magnetically ordered state is greater than in the paramagnetic (PM) region.^14−18^ For Fe_1_Ti_2_S_4_, Baranov et al.^15^ found a substantial reduction of the electrical resistivity under an applied field and at temperatures below the magnetic ordering temperature, which is concomitant with the appearance of an AFM ordering. In addition, a large MR effect with |Δρ/ρ| values up to 27% was observed in the region of a metamagnetic phase transition to the ferromagnetic (FM) state. This evolution of the electrical resistivity with temperature suggests a high sensitivity of the conduction electron scattering in Fe_1_Ti_2_S_4_ to the orientation and periodicity of Fe magnetic moments.^15^ This MR behavior was shown to be dependent on the Fe-content in Fe_*x*_Ti_2_S_4_, in which the Fe partial occupancy favored new magnetic states, such as magnetically inhomogeneous cluster-glass or granular magnetic configuration.^14^

The AHE, as reported in Fe_*x*_Ta_2_S_4_, is an important and rather rare effect, which describes the presence of a finite Hall resistance in the absence of an applied magnetic field *H*.^13^ This is considered to be a phenomenon characteristic of the metallic ferromagnets, being originated in the interplay of a finite magnetization and spin–orbit interaction.^19^ Conventionally, the Hall resistivity in an FM material is given by the relation ρ_*yx*_ = *R*_0_μ_0_*H* + ρ_*yx*_^*A*^, where *R*_0_ is the ordinary Hall coefficient, *H* is the magnetic field, and ρ_*yx*_^*A*^ is the anomalous Hall resistivity.^20^ Fe_*x*_Ta_2_S_4_ shows a Hall conductivity of ∼180 Ω^–1^·cm^–1^ below 50 K,^13^ but the origin of this phenomenon is not yet fully understood. Recently, a large AHE with a Hall conductivity of 27 Ω^–1^·cm^–1^ has been reported in CoNb_3_S_6_,^21^ which is exceptionally large compared to the small FM moment. This AHE was attributed to either the formation of a complex magnetic domain or an interplay between the magnetic domains and the electronic band structure. Other scenarios for the origin of the AHE have been also reported, including the noncollinear AFM structures and emergent time-reversal symmetry breaking.^22,23^ For instance, Thakur et al.^24^ demonstrated that the sulfide Co_3–*x*_Ni_*x*_Sn_2_S_2_ FM semimetal has multiple effects on magnetic and transport properties, exhibiting a giant coercive field of 1.2 T (for the Hall conductivity) and a significant anomalous Hall conductivity of ∼500 Ω^–1^·cm^–1^. This is observed only when the external magnetic field induces a small moment along the out-of-plane or *c*-axis, although the nature of the AHE in Co_3_Sn_2_S_2_ remains intrinsic upon Ni substitution. Nevertheless, Wang et al.^25^ showed consistently, with experimental band structures and first-principles calculations, that the intrinsic AHE in Co_3_Sn_2_S_2_ originates from the existence of magnetic Weyl Fermions near the Fermi energy level (*E*_*F*_). Thus, the origin of AHE in sulfide magnetic materials is one of the most intriguing aspects of condensed matter physics and remains controversial.

In this work, we focus on the description and the mechanisms for the AHE and its variation with composition in Fe_*x*_Ti_2_S_4_ intercalation sulfides (*x* = 0.24, 0.32, and 0.42). For this purpose, we studied the local atomic structure in these materials using X-ray absorption spectroscopy (XAS) as a function of temperature, which allowed tracking possible local structural fluctuations at the vicinities of the magnetic transition temperature. In fact, the extended X-ray absorption fine structure (EXAFS) spectroscopy provides information on the local thermal expansion and static disorder in solids, molecules, and noncrystalline materials,^26,27^ which would be useful for further insights on the magnetic properties and lattice dynamics in these sulfides. We also performed measurements of the MR and Hall effect for specific Fe_*x*_Ti_2_S_4_ members as a function of pressure and temperature, which allowed us to evaluate their magnetotransport properties. Finally, we extracted information on the pressure and temperature-dependent magnetic behavior in these compounds, using magnetic calorimetry, pressure- and temperature-dependent magnetometry, and magnetotransport measurements.

## 2 Methodology

### 2.1 Sample Preparation

Fe_*x*_Ti_2_S_4_ intercalation compounds were formed and stabilized at high pressure using our established synthesis methods described in detail elsewhere.^28^ Initially, appropriate quantities of Fe and TiS_2_ were ground, and the fine powder was inserted into a Nb-based capsule (5 mm in diameter and 15 mm in length), sealed, and then placed in a cylindrical graphite heater. We performed three syntheses using a piston–cylinder Rockland press under 3.5 GPa at 800, 850, and 900 °C for 1 h, respectively. Later, the materials were quenched and the pressure was released down to ambient conditions. As demonstrated in our previous work,^9^ the temperature plays a pivotal role in stabilizing different amounts of Fe into Fe_*x*_Ti_2_S_4_, for instance, *x* = 0.24 (900 °C), 0.32 (850 °C), and 0.42 (800 °C). From a chemical perspective, the sealed capsule avoids the volatilization of sulfur at the same time promoting the oxidation of Fe to Fe^2+^ and the reduction of Ti^4+^ to Ti^3+^.

### 2.2 Synchrotron X-ray Diffraction

The high-resolution synchrotron X-ray diffraction (SXRD) data at room temperature was performed at the ESRF beamline ID22.^29^ For this purpose, we filled a quartz-glass capillary of 0.5 mm diameter with Fe_*x*_Ti_2_S_4_ (*x* = 0.32) powder. A diffractometer with a multianalyzer stage (with 13 crystals) was used with an incident energy of 35 keV (λ = 0.35418 Å). The SXRD patterns were refined by the Rietveld method using the Fullprof software.^30^ The peak shape was described using a pseudo-Voigt function, and the full refinement included the following parameters: scale factors, zero-point error, background coefficients, asymmetry correction factors, lattice parameters, atomic positions, occupancy factors, and isotropic displacement parameters.

### 2.3 X-ray Absorption Spectroscopy

The XAS data at Fe *K*-edge (at 7.112 keV) as a function of temperature were collected at the ESRF beamline BM23^31,32^ using an unfocused beam collimated to 3(H) × 1(V) mm^2^. The monochromatic beam of two Si(111) crystals in fixed-exit geometry was obtained after the harmonics rejection from two parallel mirrors set to 3 mrad placed downstream. This configuration not only enables a precise step scanning of the monochromator of 0.3 eV at the near-edge range (XANES) and up to *k* = 16 Å^–1^ in the extended EXAFS range but also maintains the δ*k* stepping of 0.03 Å^–1^. These two regions are fundamental to address the oxidation state and geometrical arrangement (XANES), while the bond length distribution and nearest neighbors around the absorber atom are accessed in the EXAFS part. The XAS measurements were performed only in one selected member of the thiospinels series (*x* = 0.24). This sample was finely ground, mixed with cellulose, and then pelletized into disks of 5 mm in diameter to achieve an ideal absorption edge jump of ∼0.5. The measurements were performed in transmission geometry, such that the absorption coefficient was obtained from beam intensity measurements before and after the sample using two ionization chambers (30 cm in length) being filled with appropriate gas mixtures for achieving 15% (0.61 N_2_ + 1.39 He bar) and 70% (0.21 Ar + 1.79 He bar) of absorption of the photon flux, respectively. The pellet was placed inside a liquid He cryostat under a vacuum (base pressure: 10^–7^–10^–6^ mbar). The temperature was monitored using a Pt-based thermocouple. The energy drift of the monochromator was tracked by following the edge energy position of a Fe foil (≥99.95%, Goodfellow) used as a reference sample that was placed behind the sample and simultaneously measured.

The EXAFS data were recorded up to 16 Å^–1^ in *k*-space in Fe_0.24_Ti_2_S_4_ and across its magnetic ordering temperature at 60–80 K. XAS data extraction and fittings were performed using two different software packages, namely, *Athena*/*Artemis*^33^ and *Larch* XAS Viewer.^34^ In both packages, the pre-edge background subtraction, edge jump normalization, and EXAFS extraction were conducted prior to the EXAFS fitting. To run the fitting, theoretical scattering paths were calculated in the framework of the *FEFF* multiple scattering path expansion,^35^ starting from the experimental structural model for Fe_0.24_Ti_2_S_4_. In this case, a monoclinic unit cell belonging to the space group *C*12/*m*1 (No. 12) was taken as a starting point by considering that Fe, Ti, S1, and S2 fully occupy their nonequivalent sites. Then, the calculated paths were adjusted to the experimental spectra by fitting the EXAFS parameters, namely, average bond distance (*d*_Γ_) and the bond variance or Debye–Waller exponent (σ_Γ_^2^). Here, the coordination numbers were kept fixed to the ones extracted from diffraction data. The amplitude reduction factor (*S*_0_^2^ ≈ 0.9882) was obtained from EXAFS fitting of the Fe foil spectrum, and was used as a fixed parameter for all the scattering paths used to fit the temperature-dependent EXAFS spectra.^26^ To fit the EXAFS data, we have used five single scattering (SS) paths split into two shells. The first shell contains the pair-bonds Fe–S_(1)_ and Fe–Ti_(1)_, while the second shell has the pairs Fe–Fe_(2)_, Fe–S_(2)_, and Fe–Ti_(2)_. The first shell comprises the bond Fe–S_(1)_ that forms an octahedral unit [FeS_6_; coordination number (*N*_Γ_) of 6], in addition to the pairs Fe–Ti_(1)_ with *N*_Γ_ of 2. For the second shell, we considered the path Fe–Fe_(2)_ with *N*_Γ_ of 2 that corresponds to the lattice parameter *b* (∼3.42–3.43 Å^9^), the path Fe–S_(2)_ with *N*_Γ_ of 6, and the path Fe–Ti_(2)_ with dodecahedral coordination. Figure S2 of the Supporting Information illustrates a sketch of all the scattering paths considered in our model. The Fourier transform (FT) of the *k*-weighted EXAFS oscillations *k*^2^χ(*k*) was applied using the Kaiser–Bessel type-window that defined *k*-and *R*-spaces to *k* = 1.5–8 Å^–1^ and R = 1.2–4.6 Å, respectively. The fitted parameters derived from both software packages provided quite similar values. Results shown here refer only to those obtained using the *Larch* XAS Viewer. In the Supporting Information file, more details on the EXAFS fitting can be found as well as the fitting results from both software packages (see Figure S1).

### 2.4 Specific Heat

The specific heat of the intercalation sulfides was measured with a physical properties measurement system (PPMS-Quantum Design, San Diego, USA) using the heat-pulse and thermal relaxation method, on pellets directly cut from as-obtained samples. The specific heat was measured in a temperature range from 2 to 300 K without (0 T) and with an externally applied magnetic field of 9 T.

### 2.5 Pressure- and Temperature-Dependent Magnetic Properties

The magnetic measurements were obtained with the MPMS-3 system (Quantum Design, San Diego, USA) in a temperature range from 1.8 to 400 K and applied magnetic fields up to 7 T. The high-pressure magnetic properties were studied in the same SQUID magnetometer using a CuBe cylindrical cell (HMD kit), allowing pressures up to 1.2 GPa. A Teflon sample tube (2.1 mm OD) and Teflon caps were used to form the high-pressure seal for the different powder samples, using a Daphne 7373 oil as pressure transmitting media. A small Sn rod (1–2 mm in length) was also inserted inside the tube as a pressure gauge. The Sn superconducting transition temperature was used for the applied pressure determination, shifting from 3.72 K (zero pressure reference) to 3.14 K for 1.2 GPa.

The temperature-dependent magnetization (without applied pressure) was measured following a high field cooled protocol: the sample was cooled down from 300 to 2 K in a 7 T applied field, then the magnetic field was reduced, and then the magnetization was measured upon warming. The FM component was estimated as the remanent moment by measuring in a very small (even zero) applied field after such a high field cooldown; the PM-like component was estimated by comparing the temperature-dependent moments measured in the highest applied fields (5, 6, and 7 T), considering the fact that the magnetic field-dependent magnetization at such high fields is already linear when measured after a high field cooldown with decreasing field.

### 2.6 MR and Hall Effect

Magnetic field and temperature-dependent resistance and Hall data were collected with the PPMS in a temperature range from 2 up to 400 K and applied magnetic fields up to 14 T. An approximate van der Pauw four probe electrical contact geometry was considered for the magnetotransport measurements, with the applied magnetic field perpendicularly oriented to the contact-plane. MR and Hall resistance were extracted symmetrically and antisymmetrically, respectively, averaging the resistance values taken in positive and negative fields in separate branches for increasing or decreasing absolute fields for maintaining the magnetic hysteresis. The approximate resistivity was estimated using the van der Pauw calculation from one symmetrized resistance (*R*_*xx*_) measurement (i.e., current passed along the longer edge of the pellet) and the sample thickness (*t*), as ρ_*xx*_ = π/[ ln (2)*R*_*xx*_*t* ], whereas the Hall resistivity was extracted as ρ_*xy*_ = *R*_*xy*_*t* from the antisymmetrized Hall resistance (*R*_*xy*_) measured by passing the current along the diagonal of the pellet.

## 3 Results

### 3.1 Synchrotron X-ray Diffraction

**Figure 1:**
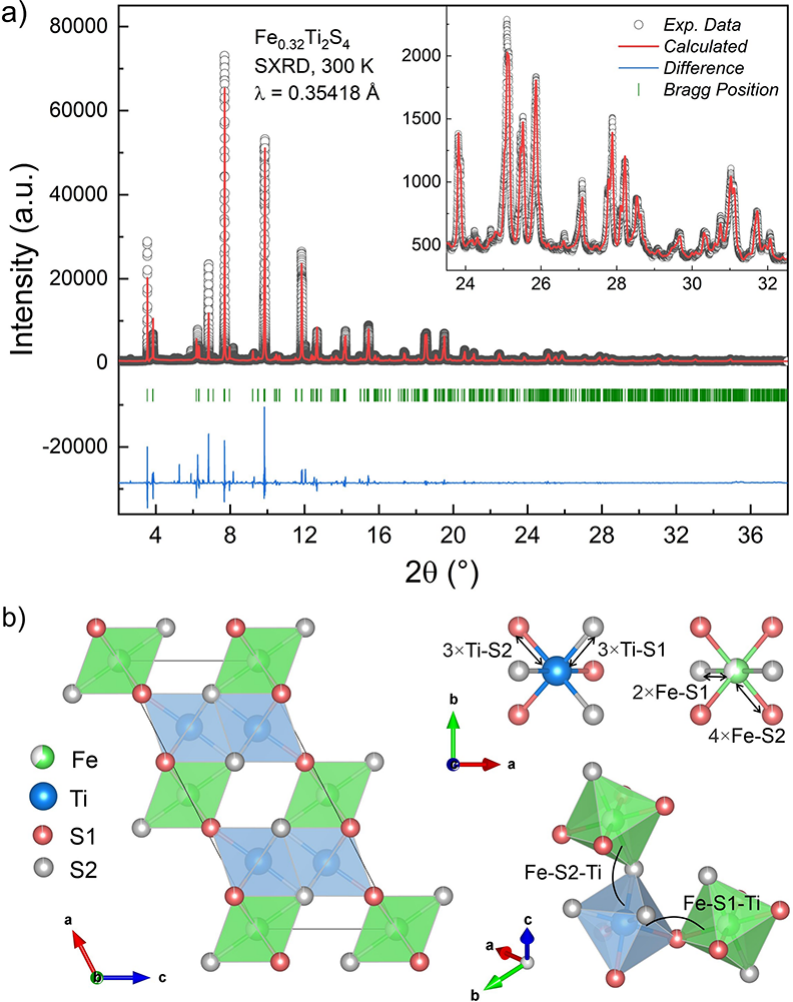
(a) Raw high-resolution synchrotron X-ray diffraction data of Fe_0.32_Ti_2_S_4_ collected at room temperature [symbols and the corresponding fit from Rietveld refinement (red curve)]. The blue curve at the bottom represents the fit residual, while green lines correspond to theoretical expected diffraction peaks. The inset displays the raw data and the refinement for high diffraction 2θ angles ∼23°–33°. (b) Crystal structure of Fe_0.32_Ti_2_S_4_ viewed along the *b*-axis (left figure) and bonding environments in the [TiS_6_] and [FeS_6_] octahedra (upper right figure) and nomenclature of angles between octahedral used in this work (lower right figure).

Figure 1a illustrates the SXRD pattern obtained at room temperature together with its Rietveld refinement for the Fe_0.32_Ti_2_S_4_ compound. The inset confirms the fitting quality for high diffraction angles in the ∼23–33° 2θ range. SXRD refinement confirmed the monoclinic crystal structure *C*12/*m*1 space group of Fe_*x*_Ti_2_S_4_ Heideite type sulfide,^9^ in which Fe atoms are located at the 2*a* (0, 0, 0) site, Ti atoms and the two types of sulfur atoms S1 and S2 are located at 4*i* (*x*, 0, *z*) sites. Figure 1b represents the crystalline structure of the Fe_0.32_Ti_2_S_4_ monoclinic Heideite. Table S1 of the Supporting Information lists the atomic positions, lattice parameters, reliability factors, and the average interatomic distances and angles obtained from the refinement. The obtained lattice parameters *a* = 12.8858(7) Å, *b* = 3.4255(9) Å, *c* = 5.9517(8) Å, and *V* = 233.60(5) Å^3^ agree with our recent Rietveld results from neutron powder diffraction (NPD) data for *x* = 0.24,^9^ even though slightly smaller. For Fe_0.32_Ti_2_S_4_, we observed very similar bond-angles between the [TiS_6_] and [FeS_6_] octahedra ⟨Fe–S1–Ti⟩ = 131.79(8)° and ⟨Fe–S2–Ti⟩ = 131.56(6)°. These octahedral units are intercalated parallel to the *bc* plane (see in Figure 1b).

### 3.2 X-ray Absorption Spectroscopy

**Figure 2:**
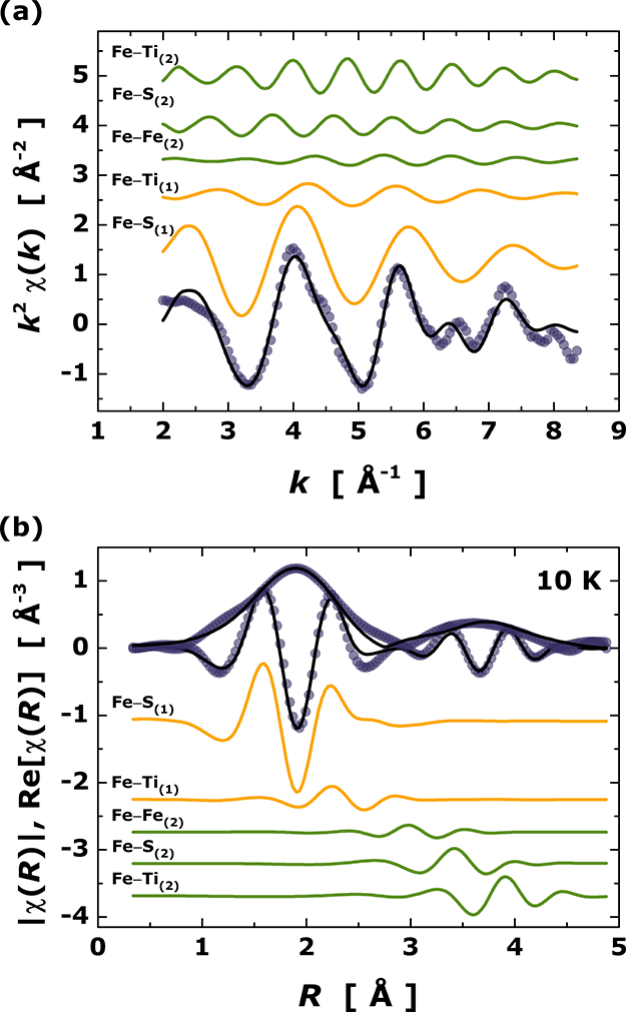
Raw Fe *K*-edge EXAFS function χ(*k*) obtained at 10 K and *k*^2^-weighted (dark blue open symbols) shown together with those of individual fitted paths (orange: first shell, dark green: second shell) and the fitted summed EXAFS signal (black line). (b) Fourier transform magnitude of *k*^2^χ(*k*) and its real part of the raw data, individual scattering paths, and summed paths [symbols and lines as in panel (a)].

In Figure 2a, a representative raw EXAFS spectrum obtained at 10 K (dark blue open symbols) is presented together with the corresponding individual signals from the fitted paths (orange and green curves) that contribute as a sum to the EXAFS function (black curve superimposed on raw data). The strong agreement of the raw data and fitted EXAFS function suggests that our model is reliable (see Figure 2a). In Figure 2b, the modulus and real part of the FT oscillations χ(*R*) in *R*-space (not corrected by photoelectron phase-shift) are shown. In Table 1, we summarized the fitted structural parameters from EXAFS at 10 K for Fe_0.24_Ti_2_S_4_ and compared them to those reported from SXRD data at room temperature. In the next step, all of the temperature-dependent EXAFS data were adjusted using the model in Table 1. The fitting convergence was stable with *r*-factors oscillating in the range 0.0297–0.0415. Table S2 of the Supporting Information lists all the fitted structural parameters for each temperature point.

**Table 1:** EXAFS Structural Parameters Were Refined from Fe *K*-Edge Spectra at 10 K for Fe_0.24_Ti_2_S_4_ Sulfide^a^

EXAFS at 10 K					SXRD at RT
SS path	*d*_Γ_ (Å)	σ_Γ_^2^ (Å^2^)	*N*_Γ_		⟨*d*⟩ (Å)
(First shell)					
Fe–S_(1)_	2.404 (6)	0.018 (1)	6		2.50 (1)
Fe–Ti_(1)_	2.85 (1)	0.019 (2)	2		2.90 (1)
(Second shell)					
Fe–Fe_(2)_	3.48 (4)	0.0185 (6)	2		3.4274 (5)
Fe–S_(2)_	4.143 (6)	0.0178 (1)	6		4.24 (2)
Fe–Ti_(2)_	4.45 (1)	0.019 (3)	12		4.46 (2)
*r*-factor	0.0334				
Δ*k* (Å^–1^)	6.5	Δ*R* (Å)	3.4		
*N*_idp_	15	*N*_v_, pts	7, 220		

a Abbreviations: *d*_Γ_ concerns the path distance, σ_Γ_^2^ the Debye–Waller exponent, *N*_Γ_ the coordination number (fixed parameter), and the ⟨*d*⟩ average path distance estimated from SXRD data at room temperature.

**Figure 3:**
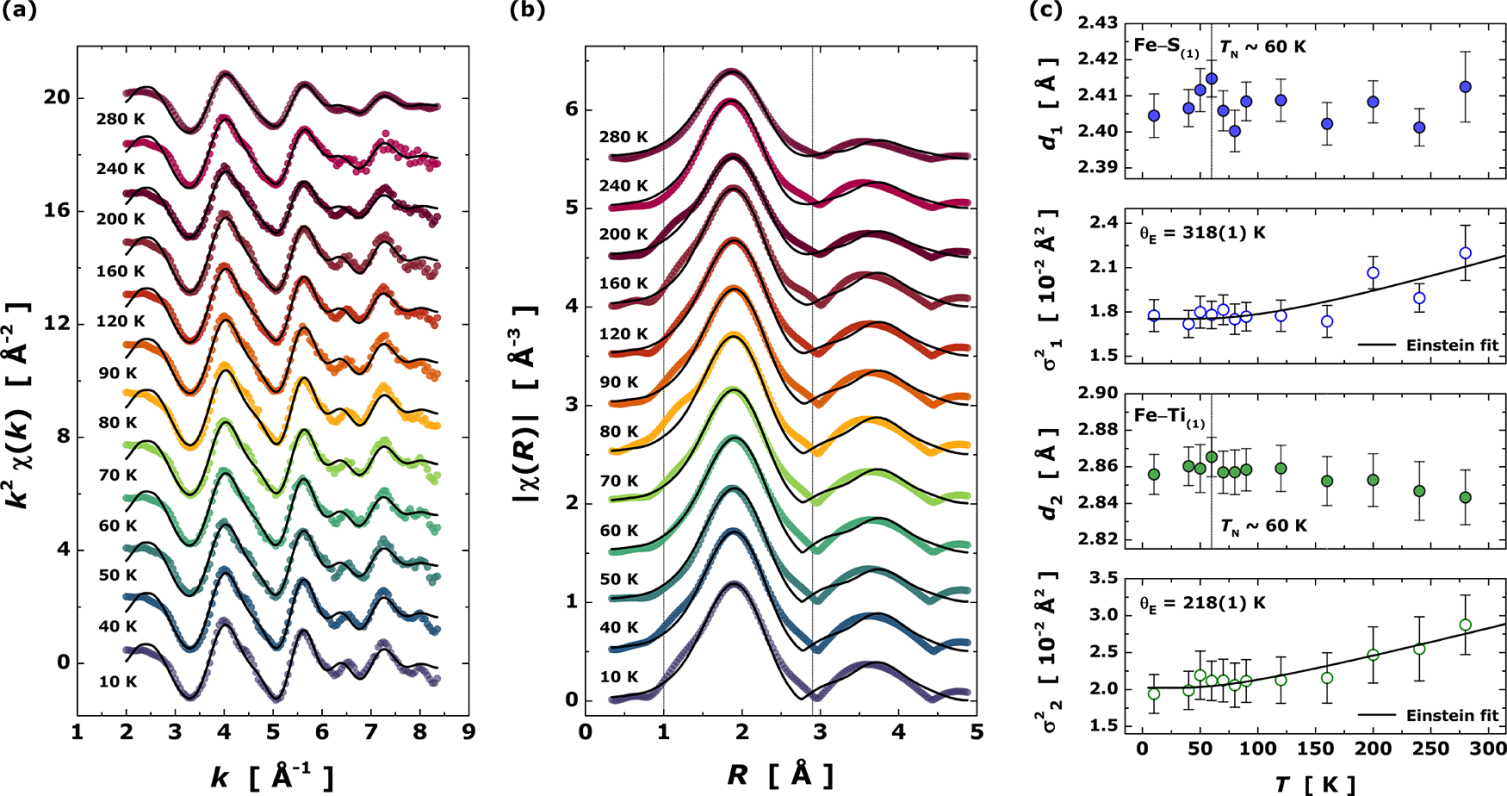
Temperature-dependent EXAFS data at the Fe *K*-edge. *k*^2^-weighted EXAFS oscillations in *k* space (a). Moduli of Fourier transform oscillations χ(*R*) in *R* space (b). Open symbols represent the raw experimental data recorded under heating 10–280 K, while the solid black lines the best EXAFS fit. Temperature dependence of the structural parameters of the first shell [Fe–S_(1)_: *d*_1_, σ_1_^2^; Fe–Ti_(1)_: *d*_2_, σ_2_^2^], obtained from the EXAFS fitting (c). The black solid lines denote the best fit of the Debye–Waller exponent to Einstein’s model. The vertical black lines in panel (c) refer to the Neel temperature (*T*_N_) at 60 K.

Considering the structural model discussed above, we probed the local structure around the Fe atom by tracking the average bond distance (*d*_Γ_) and the Debye–Waller exponent (σ_Γ_^2^) in the temperature range 10–280 K. In this way, XAS spectra were recorded with fine temperature steps of Δ*T* = 10 K near the magnetic ordering transition (*T*_N_). In Figure 3, the *k*^2^-weighted raw oscillations *k*^2^χ(*k*) (a) and the moduli of χ(*R*) (b) as a function of temperature are represented. We observed only slight continuous variations in the EXAFS function with temperature beyond a *k* of 6 Å^–1^, while in the vicinity of the magnetic transition at 60–70 K no drastic changes are observed. Similarly, we observed only slight continuous variations with increasing temperature in the shape of the moduli, in particular, the broadening of the main peak at ∼1.9 Å that was assigned as the first shell and composed by the paths Fe–S_(1)_ and Fe–Ti_(1)_.

The fitted EXAFS structural parameters for the first shell versus temperature are plotted in Figure 3c. We observed anomalies in the temperature evolution of the path distance of Fe–S_(1)_ and Fe–Ti_(1)_ (*d*_1_ and *d*_2_, respectively) around the magnetic transition temperature 60–70 K. Upon heating and just before 60 K (vertical black lines in Figure 3c), we noticed a continuous increase of *d*_1_, in agreement with a bond-distance elongation with increasing temperature. At 60 K and up to 80 K, the path distance of Fe–S_(1)_ contracts by ∼0.6%. Above 80 K, *d*_1_ increased slightly again and stayed constant within uncertainties up to 280 K. The path distance *d*_2_ Fe–Ti_(1)_ also shows a slight variation that is less pronounced than for *d*_1_. Beyond 60 K, *d*_2_ stays almost constant with increasing temperature up to 120 K. Above 120 K, *d*_2_ shows a slight contraction, indicating a negative thermal expansion. The Debye–Waller exponent for both paths shows no anomalies in the vicinity of the magnetic ordering transition that would exceed the fitting uncertainties of the individual values.

### 3.3 Specific Heat Studies

**Figure 4:**
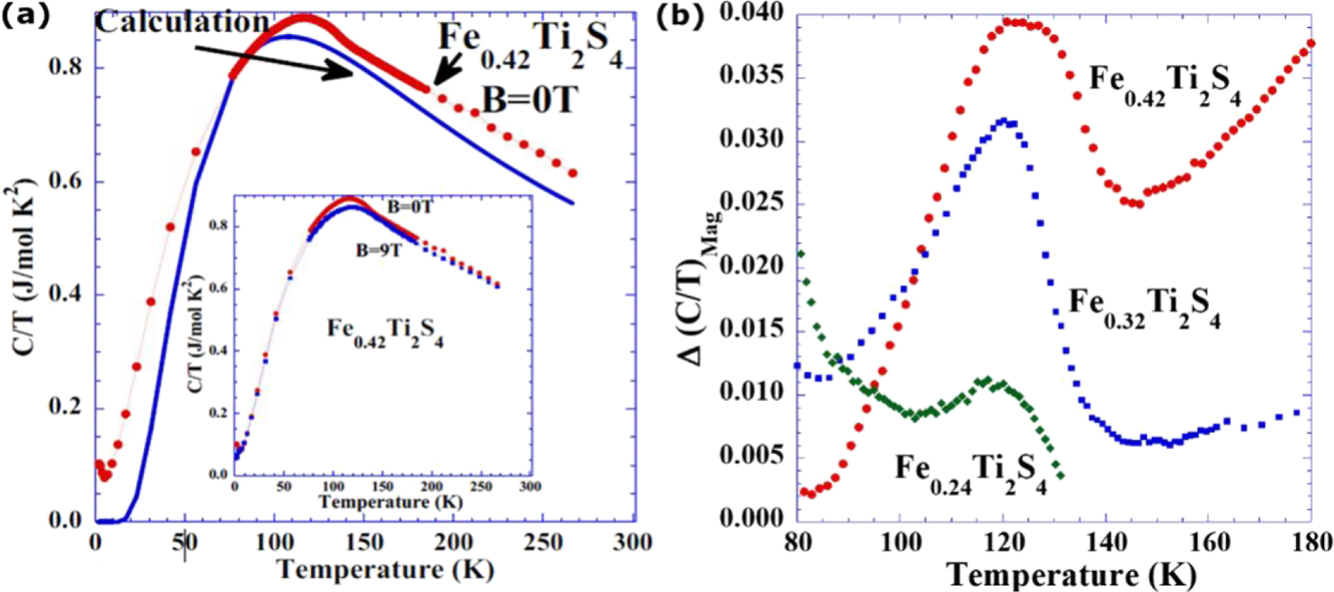
(a) Temperature dependence of the specific heat *C*/*T* for Fe_0.42_Ti_2_S_4_ and the phonon contribution calculated by assuming four harmonic Einstein oscillators at frequencies equivalents to Einstein temperatures (θ_E_) of 56(1), 190(1), 290(1), and 430(1) K. The inset shows the temperature dependence of *C*/*T* under two different applied magnetic fields (0 and 9 T). (b) Temperature dependence of the extracted magnetic component Δ(*C*/*T*)_mag_ for Fe_*x*_Ti_2_S_4_ (*x* = 0.24, 0.32, and 0.42).

We measured the temperature dependence of the specific heat for Fe_*x*_Ti_2_S_4_ (*x* = 0.24, 0.32, and 0.42) in order to investigate their magnetic phase transitions. In Figure 4a, we present the temperature dependence of the specific heat for Fe_0.42_Ti_2_S_4_ to illustrate the least-squares fitting using four harmonic Einstein oscillators centered at frequencies equivalent to the Einstein temperatures (θ_E_) of 56(1), 190(1), 290(1), and 430(1) K. These oscillators represent the lattice component of the specific heat in the range 50–300 K. After the subtraction of the lattice (phononic) contribution, one may notice a clear and broad peak around 110–120 K. We know that Fe_0.42_Ti_2_S_4_ undergoes an AFM order at *T*_N_ = 114 K,^9^ but, immediately below, there is an induced FM ordering with the application of an external magnetic field. The original AFM ordering is not recovered until the temperature increases to well above *T*_N_. In a similar manner, we also measured the specific heat under a strong applied external magnetic field of 9 T (see the inset of Figure 4a). The specific heat peak associated with the magnetic transition vanished because the entropy related to the FM-like state (under a magnetic field of 9 T) is spread over a wide range of temperatures. No clear ordering temperature is observed, and, in particular, no anomaly is observed at *T*_N_ = 114 K.

### 3.4 Magnetic Properties under Hydrostatic Pressure

**Figure 5:**
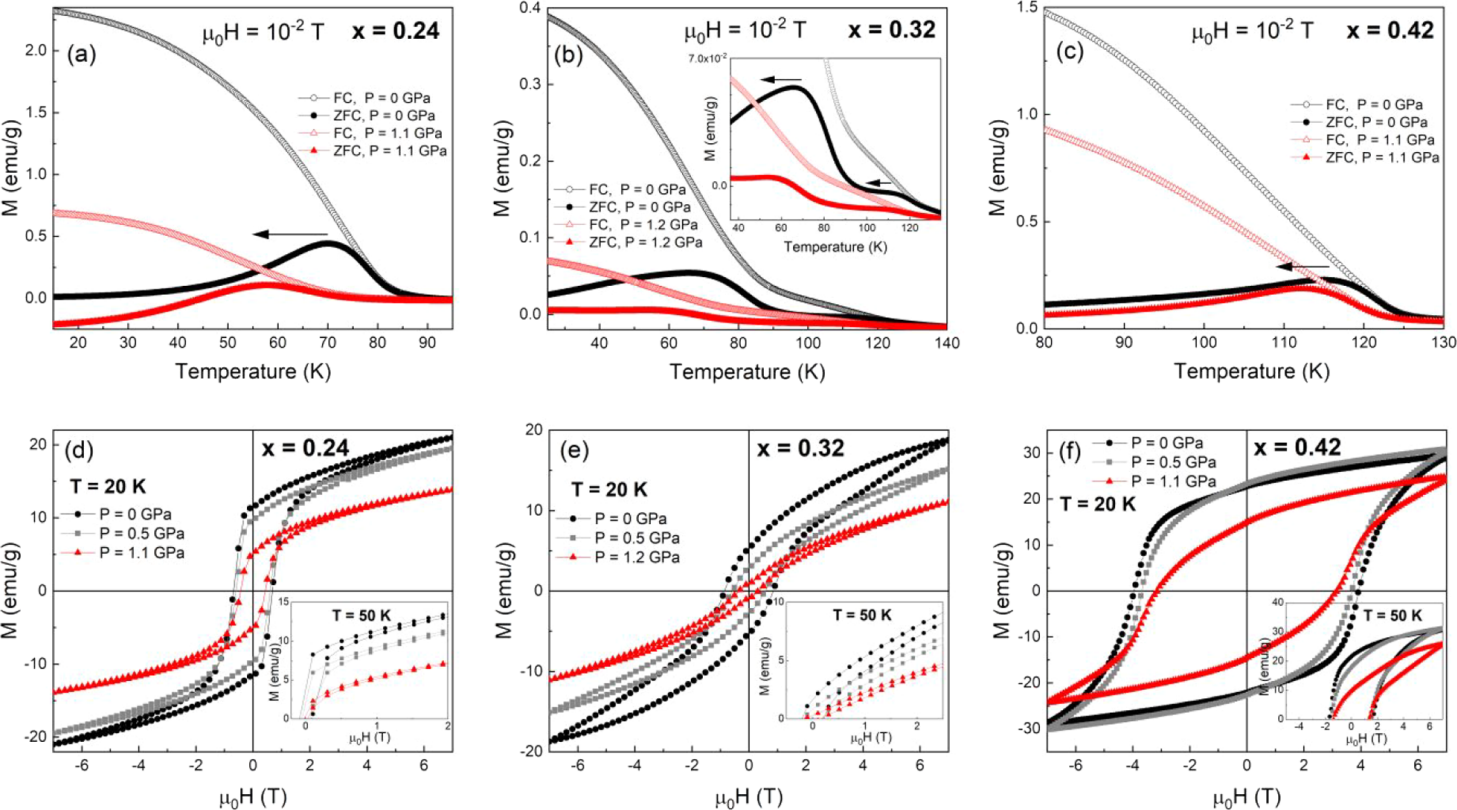
Temperature dependence of the magnetic susceptibility (a–c) and magnetic field dependence of the magnetization at *T* = 20 K (d–f), at different applied external pressures (0, 0.5, and 1.2 GPa) for Fe_*x*_Ti_2_S_4_ (*x* = 0.42, 0.32, and 0.24).

Although the magnetic properties of Fe_*x*_Ti_2_S_4_ compounds were previously described in detail,^9^ we are now interested in the nature and strength of the magnetic exchange interactions between Fe^2+^ and Ti^3+^, which could be FM-like (Fe–S–Ti) or AFM (Fe–S–Fe, Ti–S–Ti). We recall that the magnetic properties stemming from Fe^2+^ and Ti^3+^ spins offer a complex scenario with antiferromagnetic interactions, characterized by a strongly negative Weiss constant (e.g., θ_W_ = −398 K for *x* = 0.42), predominant for the Fe-rich phase Fe_0.42_Ti_2_S_4_, combined with FM-like interactions as *x* decreases (e.g., θ_W_ = 204 K for *x* = 0.24), leading to spin-glass or cluster-glass behaviors.^9^ To shed some light on this problem, we performed high-pressure and low-temperature experiments to evaluate the magnetic susceptibility and magnetization in a range of pressures (up to 1.2 GPa) and temperatures (2–150 K). The temperature-dependent magnetic susceptibility data for the three samples at three different pressures (0, 0.5, and 1.1 GPa) are presented in Figure 5a–c.

In Figure 5d–f, the hysteresis loops up to 7 T for the three samples of Fe_*x*_Ti_2_S_4_ (*x* = 0.24, 0.32, and 0.42) at three different pressures (0, 0.5, and 1.2 GPa) are shown. At *T* = 20 K, the signal coming from the CuBe HP-cell and the Sn-manometer is almost negligible. Considering only the saturation magnetization (*M*_s_), as extrapolated from the high magnetic fields, the reduction of *M*_s_ is approximately 30% for Fe_0.42_Ti_2_S_4_, depicting the strongest AFM interactions. For Fe_0.24_Ti_2_S_4_, the *M*_s_ reduction is more significant, near 50% at the same temperature and in the same pressure range. The *M*_s_ reduction is even higher, close to 90% for Fe_0.32_Ti_2_S_4_, due to the more pronounced metastable phase mixture. In fact, the major effect of the hydrostatic pressure on the exchange interactions concerns the reduction of saturation magnetization at a fixed temperature.

### 3.5 MR and Hall Effect

**Figure 6:**
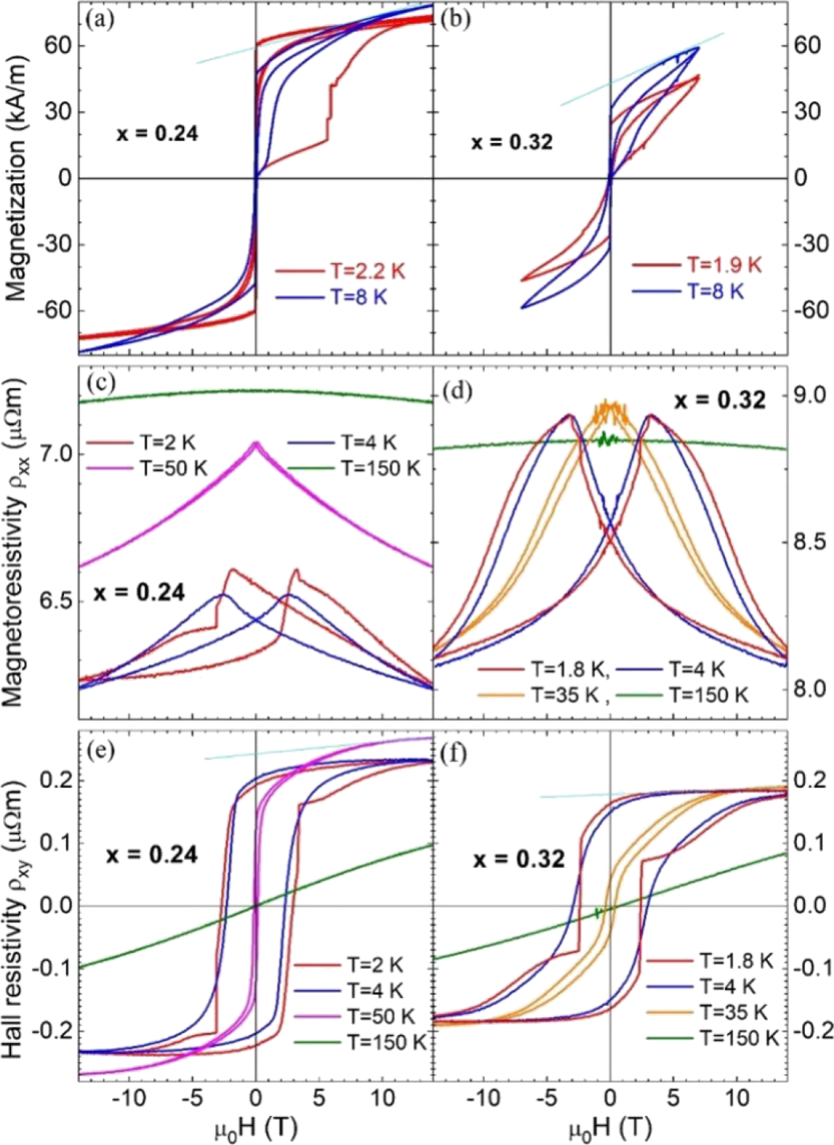
(a,b) Magnetization at *T* ≈ 2 K (red) and 8 K (blue) with the virgin curves after zero-field cooling (thick) and posterior hysteresis loops (thin). (c,d) Magnetoresistivity ρ_*xx*,_ and (e,f) Hall resistivity ρ_*xy*_ at a few illustrative temperatures. Linear fitting was performed to the high field parts of magnetization and Hall resistivity (light blue lines at selected temperatures).

The magnetization and MR of the high-pressure synthesized *x* = 0.24 and 0.32 polycrystalline samples are exhibited in Figure 6. The MR and Hall resistance were extracted by symmetrizing and antisymmetrizing the resistance measurements taken in an approximate van der Pauw configuration up to 14 T in positive and negative fields.^36^ As a main observation, pronounced jumps at very low temperature (*T* = 2 K) of the MR and magnetization can be noticed.

In Fe_0.24_Ti_2_S_4_ at *T* ≈ 2 K, sharp jumps are noticed in both magnetization and Hall resistivity in Figure 6a,e. In particular, at 1.8 K and 2 T, these sudden jumps in the magnetization reached ΔM = 20.5 kA·m^–1^ (Figure 6a). In the Hall data, at 1.8 K and 3.3 T, there is a jump in the resistance of 0.23 mΩ. With a sample thickness of 0.45 mm, the corresponding jump of the Hall resistivity is Δρ_*xy*_ = 0.1 μΩ·m (Figure 6e). The corresponding van der Pauw resistance is 3.2 mΩ, or resistivity of ρ_*xx*_ = 6.5 μΩ·m (Figure 6c), leading to a conductivity of σ_*xx*_ = 1.5 × 10^5^ S·m^–1^. Combined, there is a jump in the Hall conductivity of Δσ_*xy*_^AHE^ = ρ_*xy*_/ρ_*xx*_^2^ = 2400 S·m^–1^. For Fe_0.32_Ti_2_S_4_, because of its metamagnetic phase, there are several points to be considered before a precise extraction of the Hall properties, and these details will be discussed in the Discussion section.

## 4 Discussion

### 4.1 Local Atomic Structure

The present temperature-dependent EXAFS data reveal that the Fe_0.24_Ti_2_S_4_ sulfide depicted an anomalous contraction of the main Fe–S bond and, to a lesser extent, of the Fe–Ti bond with increasing temperature and at the vicinity of the magnetic transition in the 60–80 K temperature range. Although the bond contraction is small and amounts only to ∼0.6%, it is within the resolution of the EXAFS technique. We propose a magnetoelastic coupling that is at the origin of this contraction and that can likely also explain the dynamics and possible origin of the MR in these compounds. For the MR, the contraction may induce an overlap of the Fe and S electronic density states, which promotes an enhancement of the conductivity below *T*_N_.^37,38^ We did not observe anomalies in the EXAFS data and thus in the local structural arrangement for temperatures above the magnetic transition (*T*_N_). Similarly, no anomalies were detected for the Debye–Waller exponent (σ_Γ_^2^) along the entire probed temperature range that would characterize an emergence of the spin–phonon coupling, in analogy to our recent report on PrNiO_3_ nickelate.^39^ Most likely, the structural differences between the intercalation sulfide and the perovskite oxide may explain the absence of spin–phonon coupling in the former one. The former material has a layered structure, and the iron FeS_6_ octahedral units are less connected to their neighbors when compared to the perovskite oxide. In the latter, the NiO_6_ octahedra shared common vertices and the magnetic transition is indeed phonon-mediated.^40^ These structural differences may explain the occurrence of the mentioned magnetoelastic coupling only in (Fe,Ti)-based sulfides.

The specific heat results elucidated the presence of four Einstein oscillators for the phonon contribution, with Einstein temperatures of ∼56(1), 190(1), 290(1), and 430(1) K, respectively. From the EXAFS results, we were able to assign two of these oscillators as the atomic vibrations from the path distances Fe–S_(1)_ (θ_E_ ≈ 318 K; 290 K) and Fe–Ti_(1)_ (θ_E_ ≈ 218 K; 190 K) at the first shell level. For this purpose, the dynamic component of the Debye–Waller exponent was fitted to the Einstein’s model,^39,41,42^ as given by
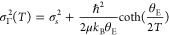
1where θ_E_ is the Einstein temperature, σ_s_^2^ represents the static disorder of the pair-bond (here, either Fe–S or Fe–Ti), while *k*_B_, *ℏ*, and *T* represent the Boltzmann constant, the reduced Planck constant, and the temperature. We extracted an Einstein temperature of 318(1) and 218(1) K and a static disorder of ∼0.014 and ∼0.016 Å^2^ for the bond-pairs Fe–S_(1)_ and Fe–Ti_(1)_, respectively. These static disorder values are considered quite high and may be due to distortions of the first shell octahedron, say to a structural origin.^43^ From the obtained Einstein temperatures, the harmonic approximation for the atomic potential can provide an estimation of the force constant (κ_E_) of the pair-bond^44−46^ as follows:
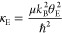
2where κ_E_ is around 3.7 eV·Å^–2^ for Fe–S_(1)_ and 2.2 eV·Å^–2^ for Fe–Ti_(1)_ meaning that the atomic interaction with sulfur is more rigid (and likely more covalent) when compared to titanium at the first shell level.

### 4.2 Spin-Glass Behavior

The spin-glass feature in (Fe,Ti)-based layered sulfide was proven by using complementary techniques, including specific heat and magnetic measurements. From specific heat, a precise lattice contribution removal allowed the characterization of only the temperature dependence of the magnetic component, as represented in Figure 4b in Fe_*x*_Ti_2_S_4_. For *x* = 0.42, the broad peak around 120 K can be assigned to AFM ordering. This peak is reduced for *x* = 0.32 and even more significantly for *x* = 0.24. We therefore propose that these latter two samples exhibit rather a cluster-glass behavior^9^ with the coexistence of a metastable phase composed of AFM and FM clusters in the temperature range from 100 to 200 K.

For higher temperatures, the magnetic results established that the cluster-glass phase may survive up to 150 K, although the FM phase rapidly disappears at temperatures around 60–80 K and coinciding with the Fe–S bond contraction (Figure 5 for *P* = 0 GPa). In addition, the total high field magnetization of Fe_0.24_Ti_2_S_4_ corresponds to 4.2 μ_B_/Fe (extrapolation to lower temperatures), of which the FM remanent magnetization accounts for ∼0.5 μ_B_/Fe, while the PM moment to ∼1.6 μ_B_/Fe and ∼2.6 μ_B_/Fe to the rest of the saturated (nonparamagnetic) magnetization. This result demonstrates that there are 5 times as many magnetic moments present in the CSG as in the FM phase.

### 4.3 Pressure Effects on the Magnetic Behavior

The role of the hydrostatic pressure field on the AFM exchange interactions (Fe–S–Fe, Ti–S–Ti) is relatively limited, and it mainly concerns the decrease of the saturation magnetization. In fact, the pressure produced a significant reduction in the FM exchange interactions (Fe–S–Ti). In Figure 5, the pressure-dependent magnetic properties in Fe_*x*_Ti_2_S_4_ (*x* = 0.24, 0.32, and 0.42) indicate that the AFM exchange interactions are significantly stronger than the FM-like exchange interactions. In detail, the high-pressure data reveal that increasing pressure significantly affects the magnetic ordering temperature. At each pressure, we extracted the ordering temperature (*T*_C_/*T*_N_) from the derivative of the magnetic susceptibility versus temperature. In the case of *x* = 0.42, the AFM ordering is established at around *T*_N_ = 114 K,^9^ while for *x* = 0.32 and 0.24 both samples present a metastable phase with AFM and FM regions equivalent to ordering temperatures (*T*_C_/*T*_N_ -like) at 74 and 82 K, respectively.^9^

**Figure 7:**
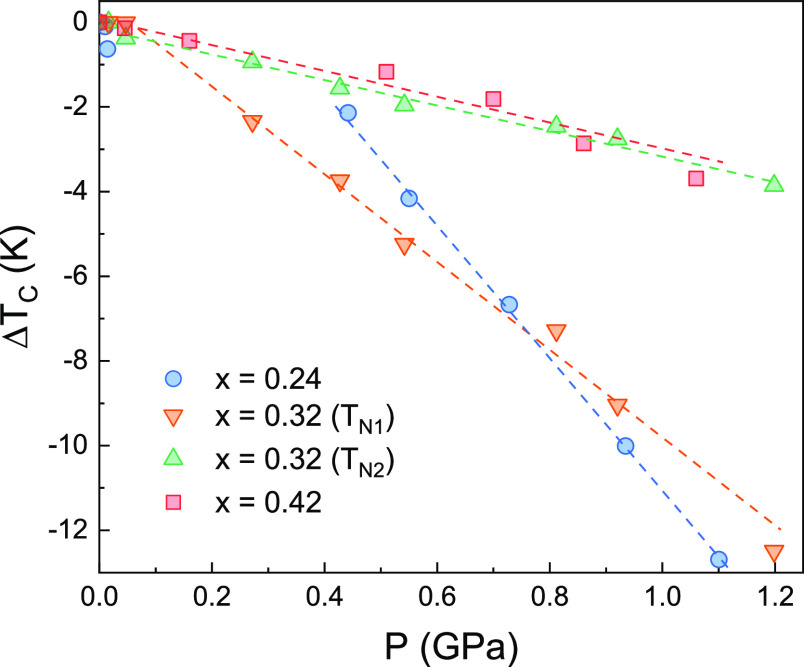
Pressure dependence of the relative variation of Δ(*T*_C_/*T*_N_), with respect to *T*_C_/*T*_N_ at *P* = 0 GPa, for Fe_*x*_Ti_2_S_4_ (*x* = 0.24, 0.32, and 0.42). Colored open symbols denote the experimental points obtained from the pressure-dependent magnetic measurements, while dashed lines are the linear best fit for extracting the slope in units of K·GPa^–1^.

The applied pressure dependence of the relative variation Δ*T*_C_/*T*_N_ (respect to *T*_C_/*T*_N_ at *P* = 0 GPa) of the magnetic ordering temperatures is presented in Figure 7. In all of the samples, the ordering temperature decreases to lower values due to the applied hydrostatic pressure. The decrease in temperature could reach up absolute values of ∼12 K (case of Fe_0.24_Ti_2_S_4_). For Fe_0.42_Ti_2_S_4_, the AFM ordering temperature (*T*_N_) decreases by ∼3 K in the full pressure range (up to 1.2 GPa). The slope of this decrease is −3.32 K·GPa^–1^. In the other end (Fe_0.24_Ti_2_S_4_), where a clear CSG state is established and the magnetic susceptibility is higher, the variation of the FM-like ordering temperature is significant, with a slope of −15.81 K·GPa^–1^. In the intermediate case (Fe_0.34_Ti_2_S_4_), a clear mixture of the two magnetic phases can be noticed (*T*_N1_ and *T*_N2_), with a clear difference in the pressure dependence of each anomaly. For *T*_N2_, its pressure dependence is close to that observed for Fe_0.42_Ti_2_S_4_, with a slope of −3.03 K·GPa^–1^. The second anomaly (*T*_N1_) is closer to that of the Fe_0.24_Ti_2_S_4_ sample, with a variation of the ordering temperature and a slope of −10.39 K·GPa^–1^. From the magnetic point of view, the Fe_0.32_Ti_2_S_4_ system seems to present a very clear metastable phase (magnetic phase mixture) with a coexistence of AFM and FM cluster domains within the sample.

### 4.4 Addressing the AHE in Fe_*x*_Ti_2_S_4_

The magnetotransport properties of Fe_*x*_Ti_2_S_4_ with 0.5 < *x* < 1.1 were reported recently by Selezneva et al.^47^ as part of a detailed magnetic NPD study. The authors identified an up to 30% decrease in the MR at the field induced AFM → FM transition in the cluster-glass state for *x* = 0.66. They also reported pronounced jumps at very low temperature (*T* = 2 K) of the MR and magnetization, similar to those observed in Figure 6, but no further details about the AHE in these compositions were provided.

Therefore, the AHE in sulfides were evaluated here by following the criteria established by the Ong’s group for MnSi^48^ and then adapted to Fe_0.5_Ta_2_S_4_.^13^ The later system consists of a layered dichalcogenide, rather similar to present compounds but based on Ta instead of Ti, which was reviewed in the seminal work on the AHE of Nagaosa et al.^20^ Their analysis is based on a careful experimental separation of the ordinary (OHE) and AHE (both magnetic field dependent) by relying on the sharp jumps in the magnetization and Hall effect, such as those observed here in Figure 6a,e at 2 K for Fe_0.24_Ti_2_S_4_. The authors also recognized the importance of the additivity of conductivities, expressing the total Hall conductivity as σ_*xy*_ = σ_*xy*_^*n*^ + σ_*xy*_^AHE^, the parallel sum of the normal or Lorentz-force ordinary Hall conductivity (OHE) σ_*xy*_^*n*^ and the anomalous Hall conductivity (or AHE) σ_*xy*_^AHE^.

In the literature on ferromagnets, the Hall resistivity can be written phenomenologically as ρ_*xy*_ = *R*_H_*B* + μ_*0*_*R*_S_*M*, where the first term is the OHE with *R*_*H*_ is the ordinary Hall coefficient, *B* is the magnetic induction, μ_0_ is the vacuum permeability, *R*_S_ is the hard-to-analyze anomalous Hall coefficient, and *M* is the magnetization. This relation can be rewritten as ρ_*xy*_*= R*_H_*B* + μ_0_*S*_H_ρ_*xx*_^2^*M* by recognizing that σ_*xy*_ = ρ_*xy*_/ρ_*xx*_^2^ as long as ρ_*xy*_ ≪ ρ_*xx*_ (here, AHE is assumed to scale linearly with the magnetization). This analysis includes the separation of the magnetization dependence of the AHE by introducing the decoupling parameter *S*_H_ = σ_*xy*_^AHE^(*H*)/*M*(*H*) to distinguish between intrinsic anomalous Hall current based on the Karpus–Luttinger theory or Berry-phase curvature, as opposed to asymmetric skew scattering off impurities and defects. The decoupling parameter *S*_H_ was shown to be constant in the FM phase of MnSi, which reinforces the idea of the intrinsic AHE. In Fe_0.5_Ta_2_S_4_, however, the anomalous Hall conductivity, and thus *S*_H_, was demonstrated to disappear more rapidly than the magnetization, with increasing temperature above 50 K, and the deviation was understood to correspond to a Hall conductivity caused by scattering from inelastic excitations, notably magnons and spin defects or domains in the uniform magnetization with opposite sign to the intrinsic anomalous Hall conductivity.^13^

**Figure 8:**
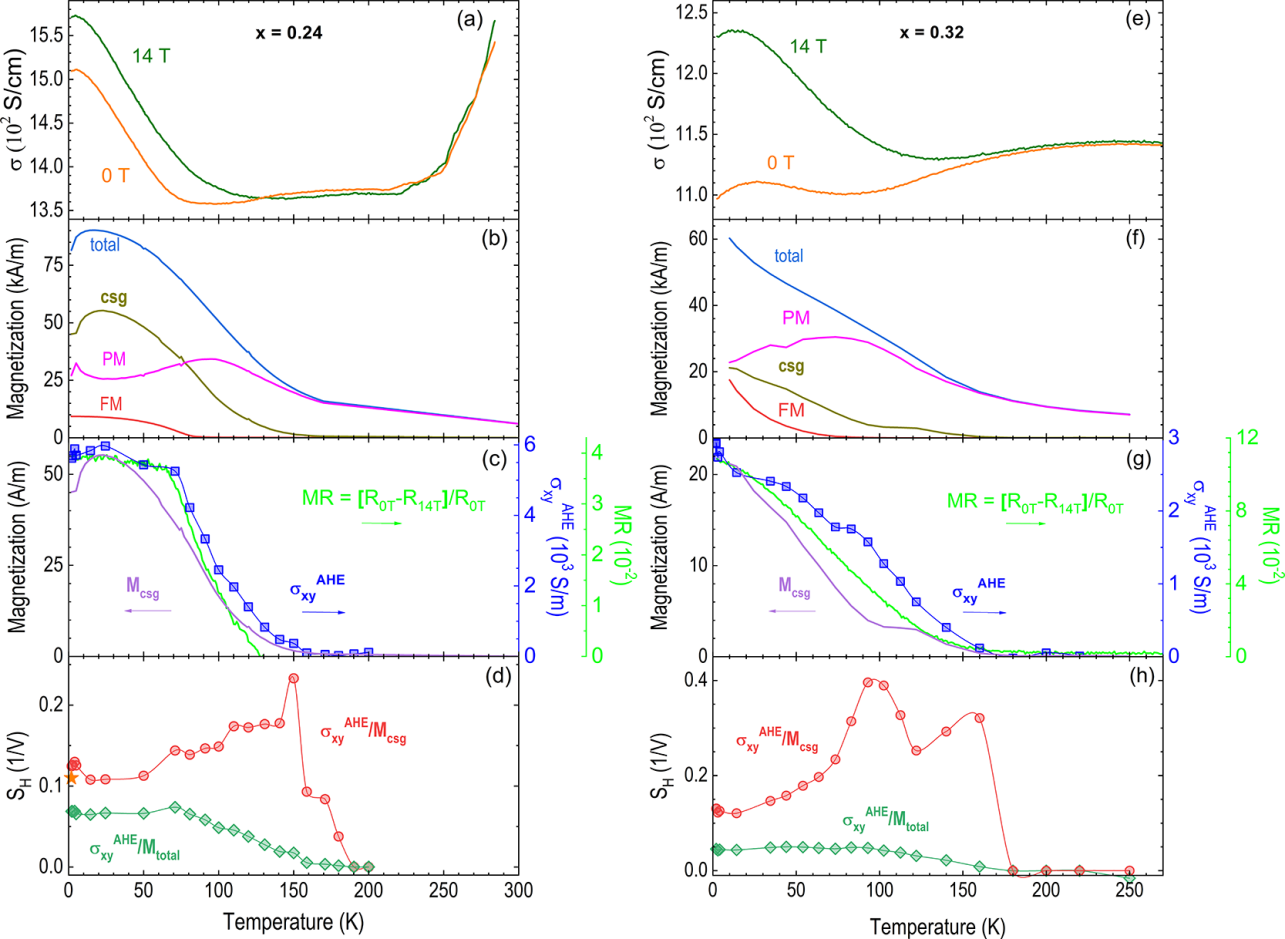
Temperature dependence of various properties of Fe_*x*_Ti_2_S_4_, with *x* = 0.24 on the left and *x* = 0.32 on the right. (a,e) Conductivity (σ_*xx*_) in 0 and 14 T. (b,f) Magnetization: total, paramagnetic, ferromagnetic, and cluster spin glass contributions. (c,g) Magnetoresistance calculated as *MR* = [*R*_0T_ – *R*_14T_]/*R*_0T_ on the green scale and anomalous Hall conductivity (σ_*xy*_^AHE^) on the blue scale and *M*_csg_. (d,h) *S*_H_ = σ_*xy*_^AHE^/*M*: with the magnetization of only the cluster spin glass and total magnetization. The orange star in panel d shows the decoupling parameter *S*_H_ extracted from the 1.8 K, 2 T jumps of magnetization, and anomalous Hall effect.

Based on the above statement, we can follow the methodology from Ong’s group to evaluate the decoupling parameter *S*_H_ = σ_*xy*_^AHE^/*M*_csg_.^13^ We may divide the anomalous Hall conductivity with the magnetization of only the CSG, because its temperature dependence matches both that of the AHE and the MR (Figure 8c,g), showing that the AHE in Fe_*x*_Ti_2_S_4_ corresponds to magnetic moments that can be saturated at higher field. The AHE extends well above 80 K where the FM contribution disappears; in our analysis, the use of only *M*_CSG_ or *M*_CSG_ + *M*_FM_ makes no qualitative difference. Following Ong’s group method, we can estimate the parameter *S*_H_ at the lowest temperature, where jumps in both magnetization and Hall resistivity are observed.^47^ In this way, a low-temperature parameter for the intrinsic anomalous Hall current can be defined as *S*_H_ = Δ*σ*_*xy*_^AHE^/Δ*M* = 0.12 V^–1^ at 1.8 K in Fe_0.24_Ti_2_S_4_ (orange star in Figure 8d). In this case, the magnetization jump at 1.8 K and 2 T corresponds to a change of 1.1 μ_B_/Fe, leading to *S*_H_ ≈ 2,200, although not in V^–1^ units. In Fe_0.32_Ti_2_S_4_, we observed sudden jumps in the AHE at 2 K and 2 T (see Figure 6f) but not in the magnetization.

On the other hand, in Fe_*x*_Ti_2_S_4_ the decoupling of OHE and AHE cannot be extended to higher temperatures based on high field switching for a lack thereof, neither in our high-pressure synthesized polycrystalline powder samples, nor in those studied by Selezneva et al.^47^ To overcome this situation, the decoupling parameter *S*_H_ at higher temperatures (*T* > 2 K) was evaluated by considering the high field saturated value of both the magnetization and Hall resistivity and separating/discarding the linear-in-field part, as illustrated by the straight cyan lines in Figure 6a,b and Figure 6e,f, as *M*_total_ = *M*_saturated_ + *χ*_Curie_*× H*. The magnetization of the CSG phase was estimated by subtracting both the PM and FM contributions from the total magnetization (Figure 8b,f). The PM contribution was estimated by comparing the temperature-dependent high field magnetizations at 14, 13, and 12 T for Fe_0.24_Ti_2_S_4_ and 7, 6, and 5 T for Fe_0.32_Ti_2_S_4_, where the CSG and FM moments are fully saturated. The FM contribution was measured upon warming in low field (fields from 0.01 to 0.1 T yield the same curves) after cooling to 2 K in high field (14 or 7 T). The PM contributions make up less than half of the total magnetization at low temperature but fully account for them above 150 K, where Fe_*x*_Ti_2_S_4_ displays the Curie-paramagnetism. Concerning the anomalous Hall conductivity, this parameter was calculated as σ_*xy*_^AHE^ = ρ_*xy*_^AHE^*×* σ_*xx*_^2^ from the anomalous Hall resistivity extracted from hysteresis loops [Figure 6e,f] and the conductivity [reciprocal of the resistivity ρ_*xx*_, in Figure 8a,e], where the linearly field-dependent ρ_*xy*_ is assumed to correspond to a single-band ordinary Hall effect.

Considering the previous discussions, the decoupling parameter *S*_H_ was estimated in the entire probed temperature range 1.8–250 K, as represented in Figure 8d,h, and its low-temperature values are around ∼0.12 V^–1^ for both *x* = 0.24 and 0.32, being justified by the value calculated from the sudden jumps at 1.8 K. The decoupling parameter *S*_*H*_ then steadily increases up to 0.2 and 0.4 for *x* = 0.24 and 0.32, respectively, and suddenly disappears above 150 K, i.e., along with the CSG magnetizations. Such a behavior can be interpreted in analogy with Fe_*x*_Ta_2_S_4_ by assuming a strong inelastic contribution to the AHE, of the same sign as the band structure-dependent *S*_*H*_ in the case of Fe_*x*_Ti_2_S_4_. We finally remark that the idea behind the decoupling parameter *S*_*H*_ is that it is mostly due to the band structure, as was shown for MnSi alloy, and such a temperature-independent behavior (*S*_*H*_ ∼ 0.04–0.06 V^–1^ for *x* = 0.24 and 0.32 when *T* > 150 K) can be recovered by comparing the AHE to the total magnetization that also includes the PM moments. In literature, Checkelsky et al.^13^ report a decoupling parameter *S*_H_ ≈ 20,000 in Fe_0.5_Ta_2_S_4_, and quote 70,000 for MnSi, i.e., 5–6 orders of magnitude higher than that derived for Fe_*x*_Ti_2_S_4_ thiospinels. The high values may be reconciled by using the magnetization indicated in both studies in Bohr-magneton units, though [see Figure 8d, h].

In parallel to the CSG, the AHE also persisted up to above 150 K, just as the MR and high field magnetization, elucidating that they may correspond to the CSG phase with strong AFM correlations. The conductivity, without applied magnetic field, continuously decreases up to 80 K (after an initial low-temperature peak coincident with the Fe–S bond contraction) and then starts to increase again around 250 K. The decrease at ∼80 K corresponds to the melting of the FM phase.^47^ This fact is highlighted by the considerably higher conductivity at 14 T, where the conductivity minimum is also shifted to around 100–120 K [see in Figure 8a,e] since the large applied field can maintain the moments aligned. The MR decreases from a low-temperature value of around 4 and 11% for *x* = 0.24 and 0.32, respectively, vanishing only above 150 K. Therefore, the increased conductivity at higher temperatures may be due to thermal excitation of the charge carriers, as shown by the slow decrease above 120 K of the ordinary Hall resistance. In parallel to the CSG, the AHE also persisted up to above 150 K, just as the MR and high field magnetization, elucidating that they may correspond to the CSG phase with strong AFM correlations.

## 5 Conclusions

In summary, three specimens of the Fe_*x*_Ti_2_S_4_ series have been prepared under high-pressure conditions; in the crystal structure of these intercalation sulfides of the Heideite type, Fe atoms occupy interstitial positions between layers of TiS_2_, both Fe and Ti atoms being in octahedral coordination. The magnetic properties stemming from Fe^2+^ and Ti^3+^ spins offer a complex scenario with AFM interactions, characterized by strongly negative Weiss constants, predominant for the Fe-rich phases, combined with FM-like interactions as *x* decreases, leading to the spin-glass or cluster-glass behaviors. The local atomic structure, followed by XAS as a function of temperature, allowed detection of local structural fluctuations at the vicinities of the magnetic transition temperature, by detecting anomalies in the temperature evolution of the path distances of Fe–S_(1)_ and Fe–Ti_(1)_ around the magnetic transition temperatures 60–70 K: the path distance Fe–S_(1)_ contracts by ∼0.6% for *x* = 0.24. We propose a magnetoelastic coupling that is at the origin of this contraction and that can likely also explain the dynamics and possible origin of the MR in these compounds. Lastly, we observed for Fe_0.24_Ti_2_S_4_ the conspicuous symptoms of a rarely noticed AHE (magnetic field-dependent), notably the sharp jumps in the magnetization and Hall effect at 2 K. Such a behavior can be interpreted in analogy with Fe_*x*_Ta_2_S_4_ by assuming a strong inelastic contribution to the AHE, of the same sign as the band structure-dependent *S*_*H*_ in the case of Fe_*x*_Ti_2_S_4_.

## Supporting Information Available

Structural parameters at room temperature obtained through Rietveld refinement from SXRD data; comparative plot showing the path distance values derived from EXAFS software *Larch* and *Artemis*; sketch of the single scattering paths considered to model the EXAFS oscillations; temperature-dependent EXAFS parameters refined from Fe *K*-edge spectra; and temperature-dependent XANES data at Fe *K*-edge (PDF)
