# Supplementary material for: Elucidating the Magnetoelastic Coupling, Pressure-Dependent Magnetic Behavior, and Anomalous Hall Effect in FexTi2S4 Intercalation Sulfides
Source: ACS Appl Mater Interfaces. 2023 Oct 20;15(43):50290–301. doi: 10.1021/acsami.3c12571 (PMC10722463; doi:10.1021/acsami.3c12571)
Supplement: Supplementary file 1 — am3c12571_si_001.pdf [file am3c12571_si_001.pdf]

Supporting Information

Elucidating the magnetoelastic coupling, pressure-dependent magnetic behavior, and anomalous Hall effect in $Fe_xTi_2S_4$ intercalation sulfides

Authors

Romualdo S. Silva Jr.[1], Joao E. Rodrigues[2], Angelika D. Rosa[2], Javier Gainza[1], Eva Cespedes[1], Norbert M. Nemes[1,3], José L. Martínez[1], José A. Alonso[1,*]

Affiliations

[1]Instituto de Ciencia de Materiales de Madrid (ICMM), CSIC, E-28049 Madrid, Spain.

[2]European Synchrotron Radiation Facility (ESRF), 71 Avenue des Martyrs, 38000 Grenoble, France.

[3]Departamento Física de Materiales, Universidad Complutense de Madrid, E-28040 Madrid, Spain.

[*] Corresponding author: ja.alonso@icmm.csic.es.

Rietveld refinement analysis

Table S1: Structural parameters of the $Fe_{0.32}Ti_2S_4$ sulfide at room temperature obtained through Rietveld refinement from SXRD data.

Atom	Wyckoff site	x	y	z	Occ.
Ti	4*i*	0.74739 (9)	0.00	0.74161 (2)	1.000
Fe	2*a*	0.00	0.00	0.00	0.309 (8)
S1	4*i*	0.62272 (1)	0.00	0.95803 (3)	1.000
S2	4*i*	0.12315 (1)	0.00	0.45648 (3)	1.000
**lattice parameters**		**reliability factors**		**average bonds and angles**	
a (Å)	12.8858 (7)	R_p (%)	10.2	⟨Fe – S1⟩ (Å)	2.420 (5)
b (Å)	3.4255 (9)	R_{wp} (%)	12.9	⟨Fe – S2⟩ (Å)	2.440 (2)
c (Å)	5.9517 (8)	R_{exp} (%)	1.36	⟨Ti – S1⟩ (Å)	2.484 (7)
V (Å^3)	233.60 (5)	R_{Bragg} (%)	8.49	⟨Ti – S2⟩ (Å)	2.432 (6)
β (°)	117.23 (1)	R_f (%)	4.89	⟨Fe – S1 – Ti⟩ (°)	131.79 (8)
ρ (g·cm^{-3})	3.677	χ^2	1.02	⟨Fe – S2 – Ti⟩ (°)	131.56 (6)

X-ray absorption analysis

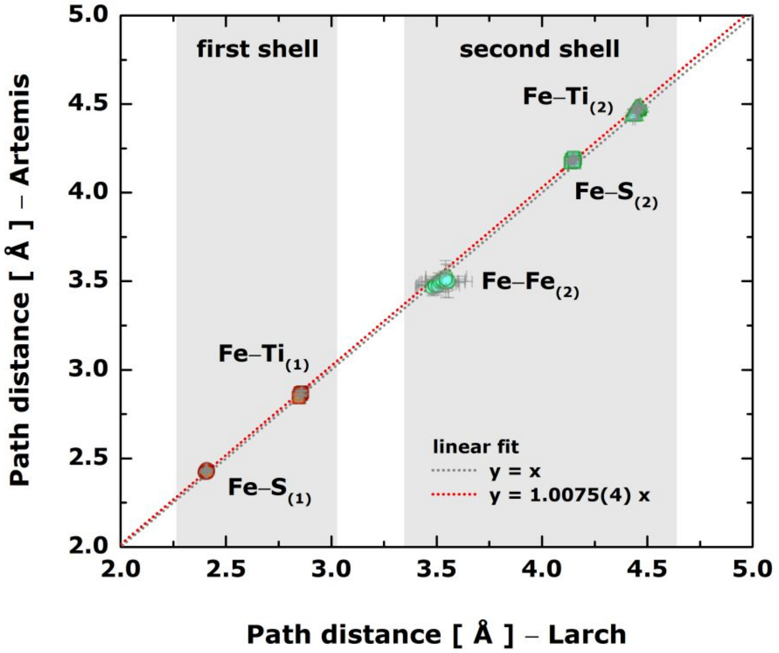

Figure S1: Comparative plot showing the path distance values [orange symbols: Fe–$S_{(1)}$, Fe–$Ti_{(1)}$; green symbols: Fe–$Fe_{(2)}$, Fe–$S_{(2)}$, Fe–$Ti_{(2)}$] as derived from both EXAFS softwares *Larch* [1] and *Artemis* [2]. The dotted gray line represents the condition that the path distances estimated by these software are equal ($y = x$). However, the linear fit (red dotted line; R^2 = 0.9998) demonstrates that the path distances from *Artemis* are in average overestimated by ~0.75(4)% when compared to those obtained from *Larch*.

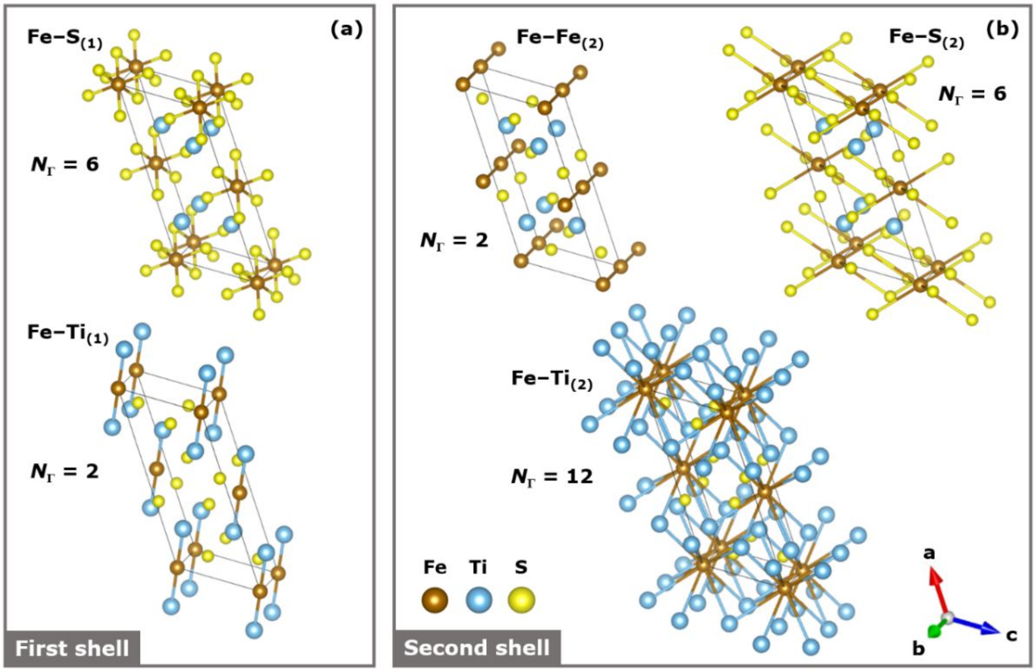

Figure S2: Sketch of the single scattering paths considered to model the EXAFS oscillations in $Fe_{0.24}Ti_2S_4$ sulfide. First shell comprises the pair-bonds Fe–$S_{(1)}$ and Fe–$Ti_{(1)}$ (a). Second shell contains the pair-units Fe–$Fe_{(2)}$, Fe–$S_{(2)}$, and Fe–$Ti_{(2)}$ (b). N_Γ stands for the coordination number.

Table S2: Temperatude-dependent EXAFS parameters refined from Fe *K*-edge spectra for $Fe_{0.24}Ti_2S_4$ sulfide using *Larch* software. d_Γ is the path distance and σ_Γ^2 the Debye-Waller factor.

	**10 K**	**40 K**	**50 K**	**60 K**	**70 K**	**80 K**	**90 K**	**120 K**	**160 K**	**200 K**	**240 K**	**280 K**
Fe–$S_{(1)}$												
d_1 (Å)	2.404 (6)	2.407 (5)	2.412 (6)	2.415 (5)	2.406 (6)	2.400 (6)	2.408 (5)	2.409 (6)	2.402 (6)	2.408 (6)	2.401 (5)	2.412 (10)
σ_1^2 (Å^2)	0.0178 (11)	0.0172 (9)	0.0180 (11)	0.0178 (9)	0.0181 (10)	0.0175 (10)	0.0177 (10)	0.0177 (11)	0.0174 (11)	0.0207 (11)	0.0189 (10)	0.0220 (19)
Fe–$Ti_{(1)}$												
d_2 (Å)	2.856 (11)	2.860 (11)	2.859 (13)	2.865 (11)	2.857 (12)	2.857 (12)	2.858 (12)	2.859 (13)	2.852 (13)	2.853 (14)	2.847 (16)	2.843 (15)
σ_2^2 (Å^2)	0.0194 (26)	0.0199 (26)	0.0219 (33)	0.0212 (27)	0.0212 (29)	0.0206 (30)	0.0211 (29)	0.0213 (31)	0.0216 (34)	0.0247 (38)	0.0255(43)	0.0288 (40)
Fe–$Fe_{(2)}$												
d_3 (Å)	3.479 (45)	3.503 (38)	3.492 (44)	3.491 (40)	3.508 (53)	3.506 (53)	3.516 (40)	3.534 (59)	3.526 (39)	3.546 (69)	3.546 (48)	3.557 (92)
σ_3^2 (Å^2)	0.0185 (68)	0.0197 (57)	0.0205 (66)	0.0198 (61)	0.0210 (82)	0.0201 (82)	0.0198 (60)	0.0223 (92)	0.0177 (58)	0.0221 (51)	0.0189 (74)	0.0252 (54)
Fe–$S_{(2)}$												
d_4 (Å)	4.143 (6)	4.147 (5)	4.155 (6)	4.161 (5)	4.145 (6)	4.136 (6)	4.150 (5)	4.150 (6)	4.139 (6)	4.150 (6)	4.137 (5)	4.137 (10)
σ_4^2 (Å^2)	0.0178 (11)	0.0172 (9)	0.0180 (11)	0.0178 (9)	0.0181 (10)	0.0175 (10)	0.0177 (10)	0.0177 (11)	0.0174 (11)	0.0207 (11)	0.0189 (10)	0.0220 (19)
Fe–$Ti_{(2)}$												
d_5 (Å)	4.452 (11)	4.459 (11)	4.457 (13)	4.467 (11)	4.454 (12)	4.454 (12)	4.456 (12)	4.457 (13)	4.446 (13)	4.447 (14)	4.438 (16)	4.443 (15)
σ_5^2 (Å^2)	0.0194 (26)	0.0199 (26)	0.0219 (33)	0.0212 (27)	0.0212 (29)	0.0206 (30)	0.0211 (29)	0.0213 (31)	0.0216 (34)	0.0247 (38)	0.0255 (43)	0.0288 (40)
r-factor	0.0334	0.0328	0.0340	0.0305	0.0322	0.0415	0.0339	0.0308	0.0332	0.0382	0.0297	0.0396
ΔE_0 (eV)	-0.93	-0.53	-0.48	-0.17	-0.60	-0.81	-0.44	-0.52	-0.85	-0.81	-1.27	-1.06

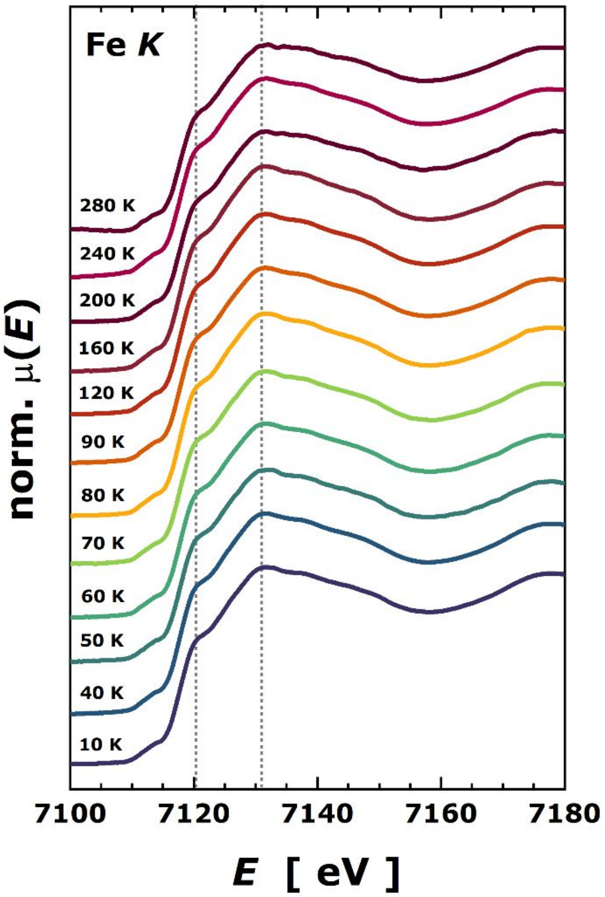

Figure S3: Temperature-dependent XANES (X-ray absorption near edge structure) data at Fe *K*-edge for the $Fe_{0.24}Ti_2S_4$ sulfide. The spectra were vertically shifted to represent the temperature evolution in the range 10−280 K.

References

(1) Newville, M. Larch: An Analysis Package for XAFS and Related Spectroscopies. *J. Phys. Conf. Ser.* **2013**, *430* (1). https://doi.org/10.1088/1742-6596/430/1/012007.

(2) Ravel, B.; Newville, M. ATHENA, ARTEMIS, HEPHAESTUS: Data Analysis for X-Ray Absorption Spectroscopy Using IFEFFIT. *J. Synchrotron Radiat.* **2005**, *12* (4), 537–541. https://doi.org/10.1107/S0909049505012719.
